# Maxillary anterior segmental distraction osteogenesis to correct maxillary hypoplasia and dental crowding in cleft palate patients: a preliminary study

**DOI:** 10.1186/s12903-023-03038-3

**Published:** 2023-05-24

**Authors:** Liwen Qian, Yufen Qian, Wener Chen

**Affiliations:** 1 Department of Orthodontics, Shanghai Ninth People’s Hospital, Shanghai Jiao Tong University School of Medicine; Collage of Stomatology, Shanghai Jiao Tong University; National Center for Stomatology; National Clinical Research Center for Oral Diseases; Shanghai Key Laboratory of Stomatology; Shanghai Research Institute of Stomatology, Shanghai, 200011 China; 2 Orthodontics, Division of Paediatric Dentistry and Orthodontics, Faculty of Dentistry, The University of Hong Kong, Hong Kong, SAR, China

**Keywords:** MASDO, Maxillary hypoplasia, Cleft lip and palate, Orthodontic treatment

## Abstract

**Background:**

The aim of this study was to present comprehensive skeletal, dental and facial aesthetic outcomes and long-term stability of maxillary anterior segmental distraction osteogenesis (MASDO) for the treatment of maxillary hypoplasia in CLP.

**Materials and methods:**

Six patients with maxillary hypoplasia treated with MASDO by a miniscrew assisted intraoral tooth-borne distractor were included. Cephalometric radiographs were obtained before distraction (T1), after the consolidation period (T2) and after orthodontic treatment or before orthognathic surgery (T3). Thirty-one cephalometric variables (12 skeletal, 9 dental, and 10 soft tissue variables) were used to evaluate changes in the dentofacial structures and the soft tissue profile. Friedman and Wilcoxon tests were applied to identify significant differences in hard and soft tissue changes during the T1–T2, T2–T3, and T1–T3 periods.

**Results:**

All patients successfully underwent MASDO without serious complications. From T1 to T2, forward movements of ANS and A (FH ⊥ N–A, VRL–ANS and VRL–A) were significant (*p* < 0.05). Significant increases in SNA and ANB were noted. Significant upward movement of points ANS (CFH–ANS) and A (CFH–A) was observed (*p* < 0.05). After distraction, a significant decrease in overjet and an increase in overbite were obtained (*p* < 0.05). Anterior tipping of the upper incisors (U1/ANS–PNS and U1/SN) was observed (*p* < 0.05). The soft tissue points of Pn, Sn, Ss, and ls showed significant anterior movement (*p* < 0.05). In addition, a significant increase in the nasolabial angle was measured (*p* < 0.05). All of the above data showed no statistically significant changes between T2 and T3 (*p* > 0.05).

**Conclusion:**

MASDO using a miniscrew assisted tooth-borne distractor presented significant maxillary advancement and favorable long-term stability in treating CLP patients with maxillary hypoplasia.

## Background

Maxillary hypoplasia is a common deformity in cleft lip and palate (CLP) patients. Skeletal class III malocclusion, a collapsed maxillary arch, dental crowding, nasolabial deformities and velopharyngeal insufficiency (VPI) are the most common clinical manifestations in these patients, which seriously affect their facial aesthetics, oral function, speech intelligibility and psychological health. Approximately 25% of these patients require orthognathic surgery for correction [1, 2].

LeFort I osteotomy (LFI) is widely used for this purpose, but poor maxillary and alveolar bone growth and palatal scarring lead to difficulty in maxillary advancement greater than 6 mm [2]. Prominent relapses have been documented in CLP patients receiving conventional LFI for maxillary advancement, especially in those with large maxillary movements [3]. Maxillary advancement disrupts the velopharyngeal structure and may aggravate or result in velopharyngeal imcompetence [4].

Distraction osteogenesis (DO) combined with Le Fort I osteotomy has been applied to correct maxillary hypoplasia since the early 1990s [5–8]. Through simultaneous elongation of the soft tissue near the distraction region, the recurrence rate of maxillary DO is markedly decreased compared with those following conventional LFI [4]. Despite its effectiveness, this method still failed to generate adequate alveolar bone in the maxillary for tooth alignment during orthodontic treatment. O’Gara and Wilson mentioned that the risk of velopharyngeal dysfunction in CLP patients and underwent maxillary DO was similar to that following conventional LFI, indicating maxillary DO may also worsen velopharyngeal function. This possible negative impact on velopharyngeal function might be due to advancement of the posterior bone fragment of the maxilla complex by pterygomaxillary disjunction during surgery [9, 10].

To circumvent the pitfalls of the abovementioned approaches, an innovative strategy was proposed to distract the anterior maxilla alone rather than the entire maxilla to correct its hypoplasia, namely, maxillary anterior segmental distraction osteogenesis (MASDO). In 2003, the first successful clinical application of MASDO using an intraoral tooth-borne distractor was reported by Dolanmaz [11]. In 2007, Iida presented a report of MASDO using intraoral bone-borne distractors with 4 mini-screws instead of tooth anchor distractor to provide effective distraction without dental effects [12]. Subsequently, clinical research using different distractors was carried out to determine the optimal protocols for MASDO [12–15]. MASDO is essential to create an alveolar bone space to alleviate dental crowding and simultaneously correct maxillary hypoplasia. Moreover, MASDO is also indicated for growing patients and might improve facial aesthetics and dental occlusion before adulthood [1].

In this study, the authors present preliminary clinical results for correction of maxillary hypoplasia secondary to CLP by advancement of the anterior maxillary segment using a miniscrew assisted intraoral tooth-borne distractor. The distraction efficacy, advantages, and long-term distraction outcomes are discussed.

## Materials and methods

This study was approved by the Institutional Review Board of Shanghai Ninth People’s Hospital affiliated to Shanghai Jiao Tong University, School of Medicine. Written informed consent was obtained from the parents.

### Subjects and methods

Six patients with CLP (5 females and 1 male; mean age, 15.2 years; age range, 14–16 years) who underwent MASDO using a miniscrew assisted tooth-borne device from January 2012 to July 2021 at the Department of Orthodontics, were enrolled and analyzed. The choice of MASDO was determined by a team including oral and maxillofacial surgeons, orthodontists, speech pathologists and periodontists irrespective of patient gender and age. Inclusion criteria: maxillary skeletal hypoplasia secondary to CLP without an alveolar cleft (submucous cleft palate, cleft of the soft palate and incomplete cleft palate); a sella-nasion-A point (SNA) angle less than 78°; a reverse overjet greater than 3 mm; age from 14 to 16 years; cervical vertebral maturation Stage (CVMS) IV and V [16]; and a collapsed dental arch and crowding in maxilla.

Before distraction (T1), after the consolidation period (T2) and after orthodontic treatment or before orthognathic surgery (T3), extra- and intraoral photographs, lateral cephalograms and panoramic images were obtained for all cases.

### Customized tooth-borne distractor design and fabrication

**Fig. 1:**
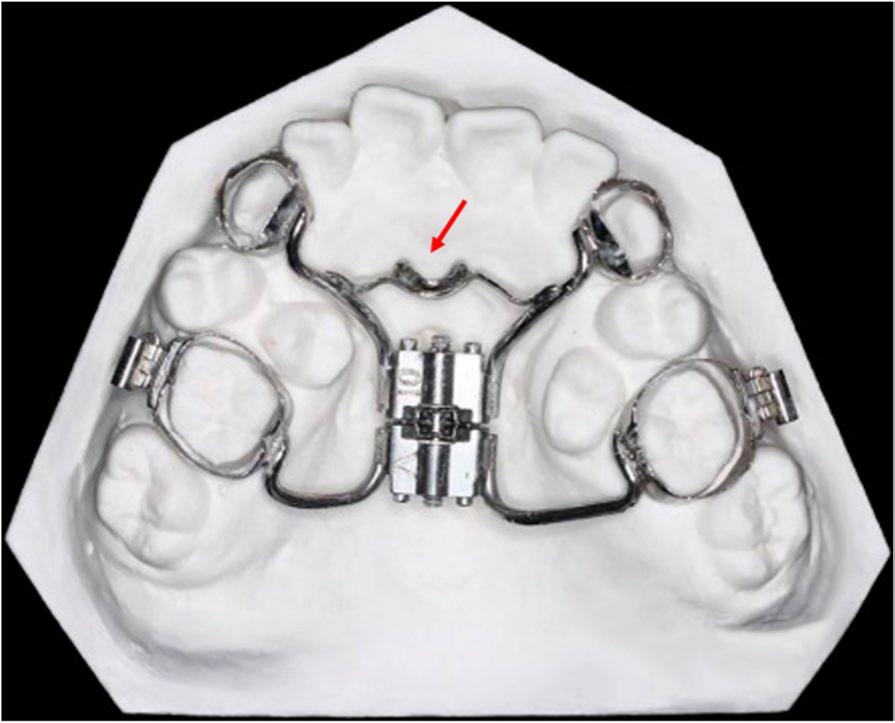
Dental cast with the intraoral tooth-borne distractor of #4. The location indicated by the red arrow is the position where a mini-screw would be implanted during surgery

The miniscrew assisted tooth-borne distraction device was custom fabricated and consisted of a Hyrax expansion screw, four orthodontic metal bands (Tomy, Tokyo, Japan) and five metallic arms (Fig. 1). Appropriate bands were selected for maxillary first molars, maxillary premolars or canines (according to the position of incision and the space assignment). With bands well positioned in the mouth, an alginate impression was obtained, and the bands were seated in the impression material. Separating elastics were placed to prevent space closure while the appliance was being constructed. A Hyrax expansion screw was placed parallel to the occlusal plane and positioned at the center of the palate. The screw was oriented in an anteroposterior direction to move the anterior maxillary segment in the sagittal plane. The expansion screw was soldered with 1.0-mm metallic arms (connective arms) close to the bands. Another arm (anchorage arm) was soldered transversely between the two anterior connective arms, the middle of which was shaped like a pocket. A mini-screw would be implanted exactly in the “pocket” against the distractor in the surgery. After implantation, the mini-screw was attached to the anchorage arm so that more expanding force was transferred to the mini-screw through the anchorage arm, which was finally applied to the premaxilla, reducing the force applied to the anterior teeth, thus enhancing the distraction effect. These positions of the “pocket” should be determined by means of three-dimensional computed tomography so that the surgeon can confirm the palatal bone shape and thickness, the location of the incisive foramen and the position of the incisor’s root. The number of “pockets” can be two to avoid the mini-screw being implanted into the incisive foramen.

### Surgery

All operations were carried out under general anesthesia by the same surgeon. Osteotomy was conducted in accordance with the planned lines. First, a complete horizontal osteotomy was performed at least 4 mm away from the dental root apex toward the edge of the piriform. Nasal septum was then separated from the nasal base was conducted. Next, the vertical osteotomy lines were marked using a small round burr and completed with a surgical saw. Buccal maxillary, lateral nasal bone, interdental, and palatal osteotomies were performed and joined with each other with the guidance of vertical osteotomies. The mucoperiosteal flap was closed after osteotomies, and the distractor was cemented to the teeth with glass-ionomer cement [12, 17]. Finally, the miniscrew (Bioray, Taiwan) was inserted in accordance with the distractor as planned.

### Distraction protocol

After a 7-day latency period, the distraction period began by turning the hyrax screw twice a day (0.5 mm/d). After a total of 14 days of distraction, the consolidation period was started and lasted for three months. The distraction protocol was referred to the study of Block et al. and Dolanmaz et al. [5, 11]. At the end of this period, the distractor was removed, and postoperative orthodontic treatment began at the same time. Notably, removal of the distractor can be performed easily without anesthesia, and the patients experienced little pain.

### Orthodontic treatment after MASDO and orthognathic surgery

After consolidation, orthodontic treatment began with a self-ligating bracket (0.022-inch slots, Damon Q, Ormco). Leveling and alignment initiated by 0.014 copper-nickel-titanium (CuNiTi, Ormco) was followed by 0.014*0.025 CuNiTi, 0.018*0.025 titanium molybdenum, and stainless-steel in arch wires. A power chain was delivered to close the residual space by MASDO. After preoperative orthodontic treatment was completed, orthognathic surgery was performed as planned.

### Cephalometric analysis

**Fig. 2:**
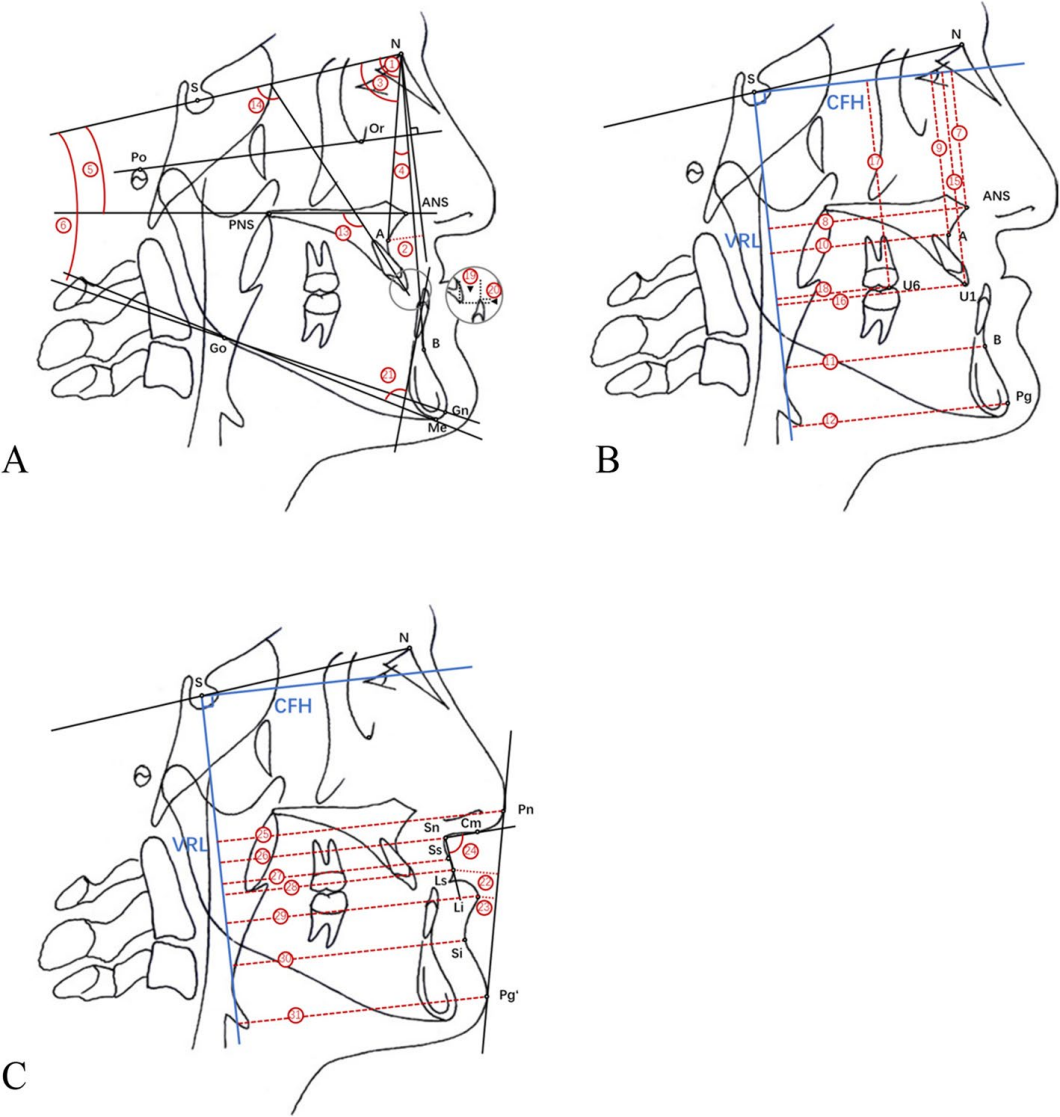
**A-C** Cephalometric variables (12 skeletal, 9 dental, and 10 soft tissue variables) used to evaluate changes in the dentofacial structures and the soft tissue profile: (1) SNA, (2) FH⊥N–A, (3) SNB, (4) ANB, (5) SN/ANS–PNS, (6) SN/Go–Gn, (7) CFH–ANS, (8) VRL–ANS, (9) CFH–A, (10) VRL–A, (11) VRL–B, (12) VRL–Pg, (13) U1/ANS–PNS, (14) U1/SN, (15) CFH–U1, (16) VRL–U1, (17) CFH–U6, (18) VRL–U6, (19) Overjet, (20) Overbite, (21) IMPA, (22) Ls–E line, (23) Li–E line, (24) Nasolabial angle, (25) VRL–Pn, (26) VRL–Sn, (27) VRL–Ss, (28) VRL–Ls, (29) VRL–Li, (30) VRL–Si, and (31) VRL–Pg′

Lateral cephalometric tracings of T1, T2, and T3 were superimposed on the SN plane at sella. The measuring coordinate system was established on the superimposition of cephalometric tracings. The horizontal reference line, the x-axis, was constructed with the Frankfurt Horizontal (CFH). This line was drawn with an angle of 7 degrees to the SN line at the sella point. A perpendicular line passing through the sella to the x-axis served as the y-axis (Vertical Reference Line, VRL) [17]. Table 1 shows landmarks and reference lines traced on the radiograph. Thirty-one cephalometric variables (12 skeletal, 9 dental, and 10 soft tissue variables) were used to evaluate changes in the dentofacial structures and the soft tissue profile (Fig. 2). All cephalometric tracings and measurements were completed by one author.

**Table 1:** Cephalometric landmarks and reference lines

Measurement	Description
S = sella	Midpoint of the sella turica
N = nasion	Most anterior point on the frontonasal suture in the middle
Or = orbitale	Most inferior and anterior point on the orbital margin
Po = porion	Upper- and outer-most point on the external auditory
ANS = anterior nasal spine	Tip of the bony anterior nasal spine in the middle
PNS = posterior nasal spine	Tip of the posterior nasal spine in the midline
A = subspinale	Deepest point on the curved profile of the maxilla between the anterior nasal spine and alveolar crest
B = supramentale	Deepest point on the curved profile of the mandible between the chin and alveolar crest
Go = gonion	Most posterior and inferior point on the angle of mandible
Gn = gnathion	Most anterior and inferior point on the bony chin
Pg = pogonion	Most anterior point on the bony chin
Me = menton	Most inferior point of the mandibular symphysis in the midline
U1	Tip of the crown of the most anterior maxillary central incisor
U6	Mesial cusp tip of the maxillary first molar
FH⊥N	A perpendicular line passing through the nasion to the Or-Po plane
U1/ANS–PNS	Axial inclination of the maxillary incisors to the ANS-PNS plane
U1/SN	Axial inclination of the maxillary incisors to the SN plane
Pn = pronasale	Most prominent anterior part of the nose tip
Cm	Most anterior point on the base of the nose (columella)
Sn = subnasale	Junction between the lower border of the nose and beginning of the upper lip in the mid-sagittal plane
Ss	Deepest point on the curved profile of the upper lip between Sn and Ss
Ls = labrale superius	Maximum convexity of the vermillion border most prominent in the mid-sagittal plane
Li = labrale inferius	Most prominent point on the vermillion border in the mid-sagittal plane
Si	Deepest point on the curved profile between Li and Pg’
Pg’ = pogonion	Soft tissue pogonion, the most prominent or anterior point on the soft tissue chin
Nasolabial angle	An angle formed between the upper lip and base of the nose (columella)
E line	A line drawn from tip of the nose to soft tissue pogonion
CFH	The horizontal reference line, the x-axis, a line drawn with an angle of 7 degrees to the SN line at the sella point
VRL	The vertical reference line, the y-axis, a perpendicular line passing through the sella to the x-axis

### Statistical analysis

Since the data did not show a normal distribution, Friedman (*p* < 0.05) and Wilcoxon (*p* < 0.05) tests were applied to identify significant differences in hard and soft tissue changes during the T1–T2, T2–T3, and T1–T3 periods. Six weeks after the first measurements, tracings and calculations were repeated by the same author.

## Results

### Patient demographics

**Fig. 3:**
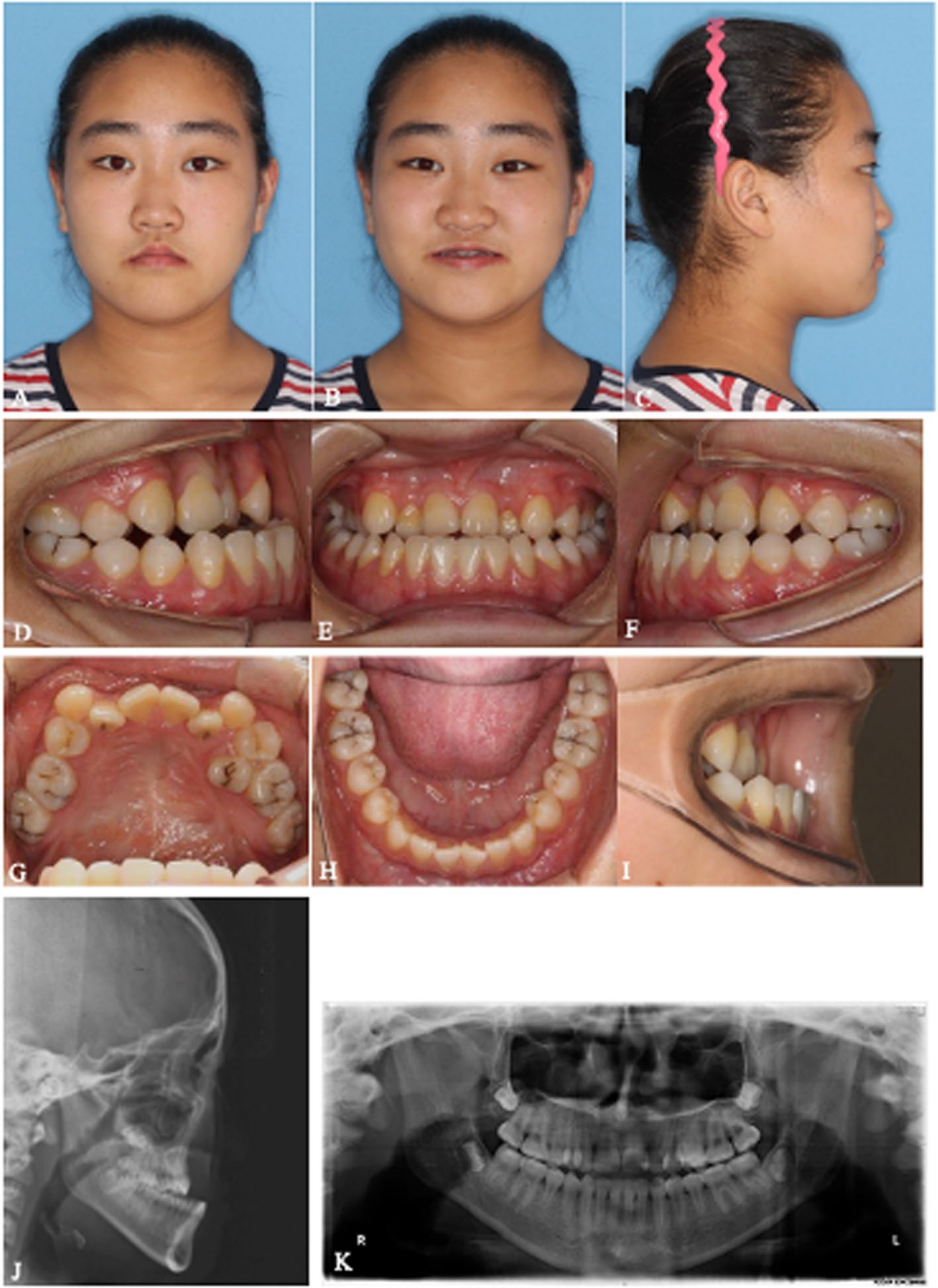
**A**-**K** Pretreatment extra- and intraoral photographs, lateral cephalograms and a panoramic image of #1. A 15-year-old Chinese girl who presented with a submucous cleft palate and without any additional clinical signs suggestive of other syndromic disease was referred to this department for correction of the skeletal discrepancy of the face. She had severe midfacial retrusion and an anterior crossbite of approximately 8 mm and was missing tooth No. 15. Severe crowding in the upper arch with no crowding in the lower arch was observed on oral examination. No. 25 was in a totally palatal position. Space to release the severe crowding in the upper arch was insufficient even if No. 25 was extracted. A cephalometric evaluation established that Class III malocclusion resulted from both maxillary hypoplasia and mandibular hyperplasia. The following treatment plan was proposed: (1) maxillary forward advancement with MASDO; (2) alignment of the malposed teeth and closure of the distracted space; (3) mandibular set back via bilateral sagittal split ramus osteotomy (BSSRO); (4) completion to achieve tight intercuspation; (5) retention

**Fig. 4:**
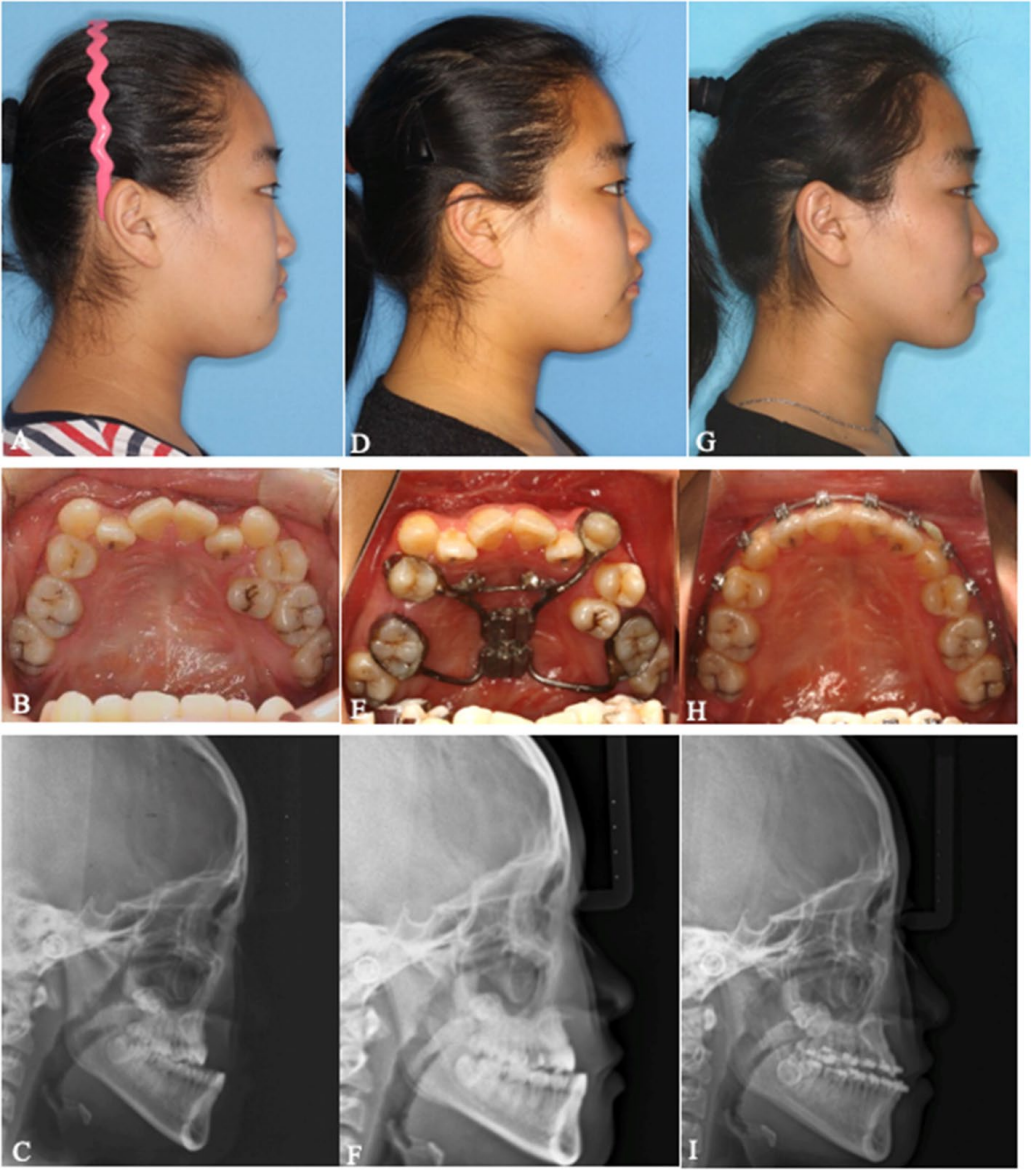
**A-C** Photographs, intraoral view and lateral cephalometric radiograph before treatment. **D-F** Photographs, intraoral view and lateral cephalometric radiograph after the consolidation period. **G**-**I** Photographs, intraoral view and lateral cephalometric radiograph after preoperative orthodontic treatment. MASDO was applied and the osteotomy line was drawn on the palate between the first molar and first premolar on the right side and canine and first molar on the left side. Two mini-screws were inserted in the planned position. The premaxilla moved forward and the facial profile was improved after MASDO. No. 25 was extracted during the orthodontic treatment and the remaining dental crowding was alleviated using the space produced by the MASDO procedure

**Fig. 5:**
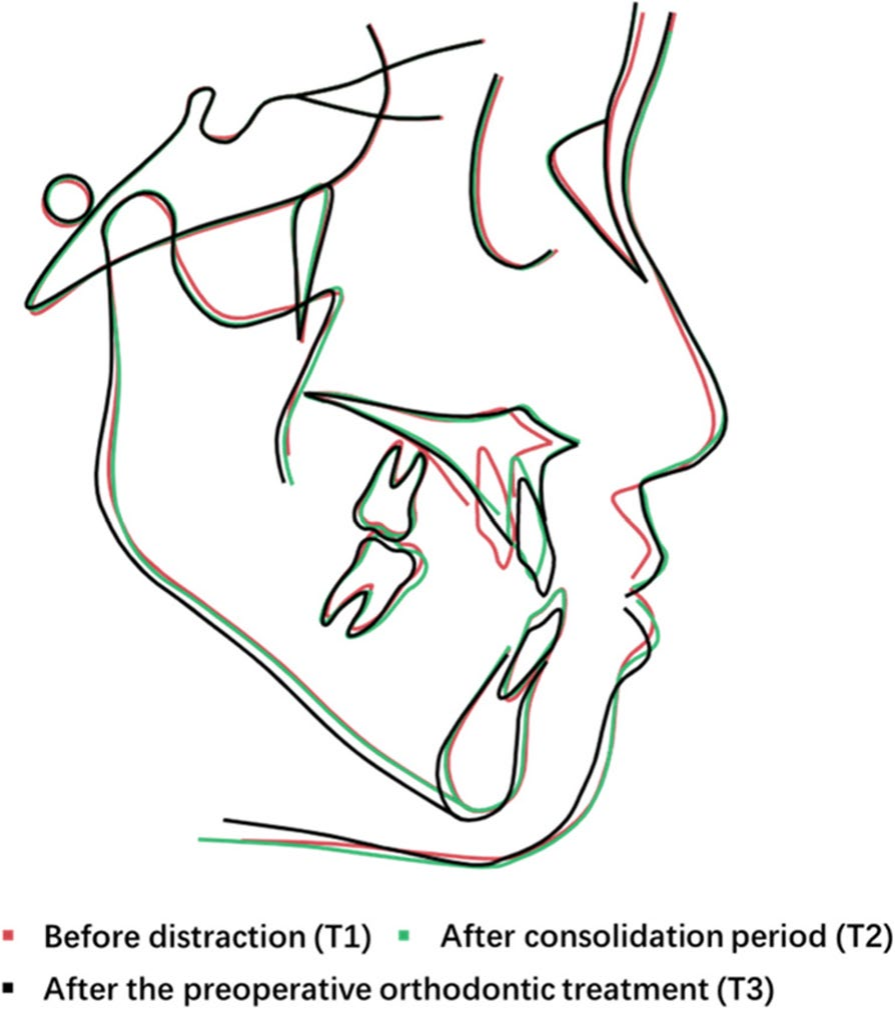
Superimposition of the profilogram on the sellanasion plane at the sella. Superimposition of cephalometric tracings of #1 made before distraction (T1), after consolidation period (T2) and after the preoperative orthodontic treatment (T3). MASDO caused significant advancement in the anterior segment of the maxilla and improved patients’ facial aesthetics with minimal relapse

**Fig. 6:**
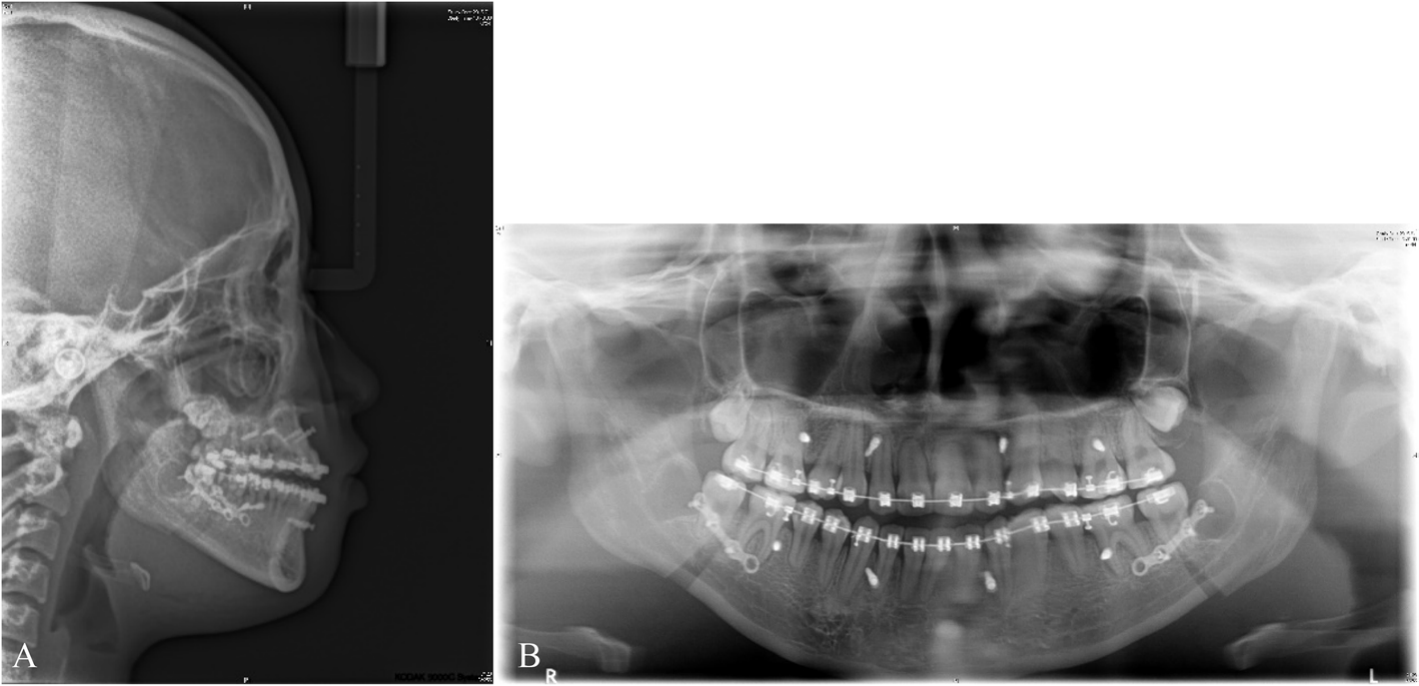
**A**, **B** Lateral cephalometric and panoramic radiographs 2 weeks after BSSRO of #1. 26 months after the MASDO procedure, BSSRO was performed to correct the patient’s mandibular prognathism and anterior crossbite. The amount of mandibular setback was approximately 4.0 mm on both sides

**Fig. 7:**
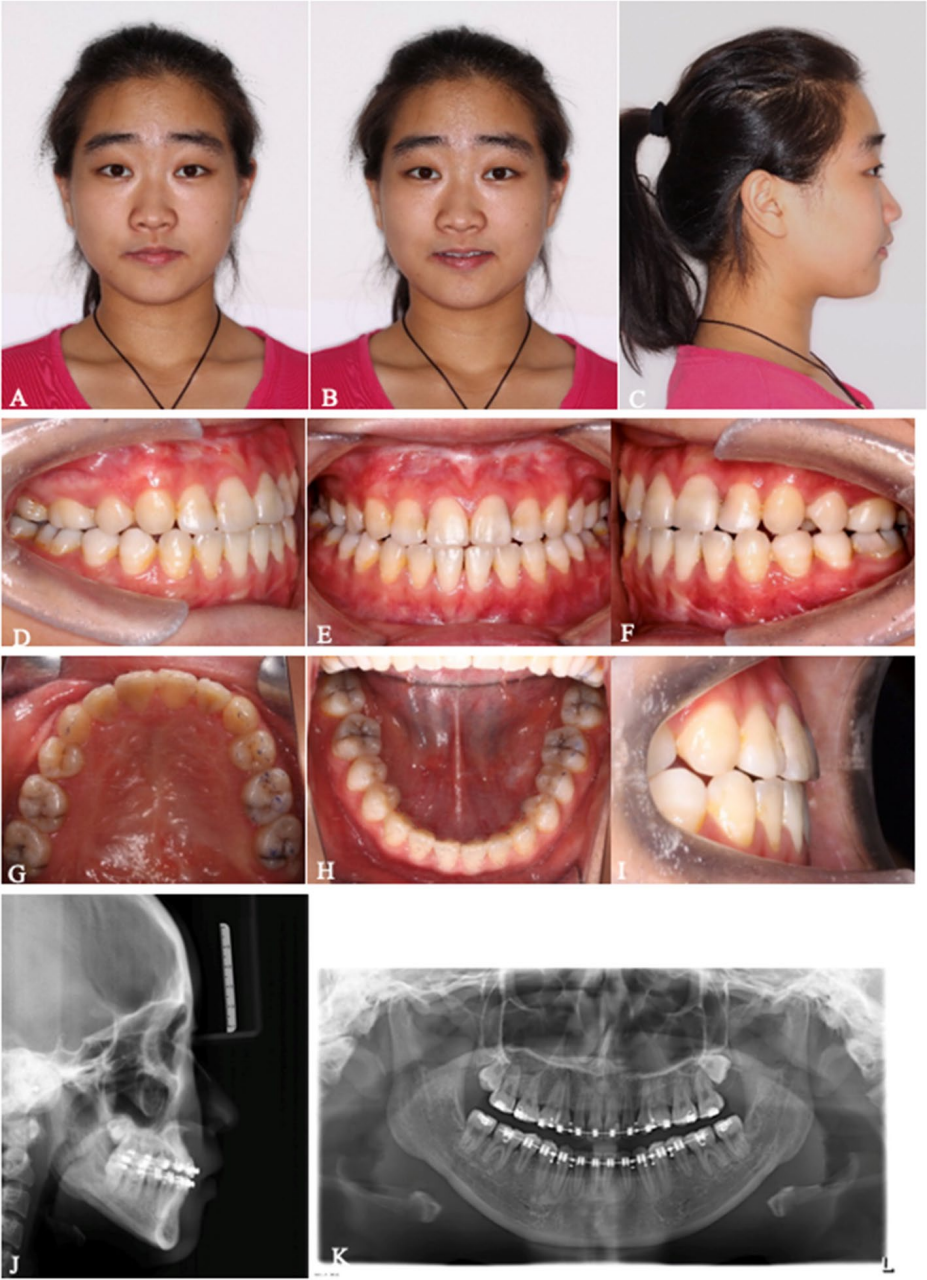
**A**-**K** Photographs, intraoral view, lateral cephalometric and panoramic radiographs after orthognathic surgery and postoperative orthodontic treatment of #1. The titanium plates were removed 8 months after BSSRO. All appliances were removed and the final X-rays were taken 12 months after BSSRO

**Fig. 8:**
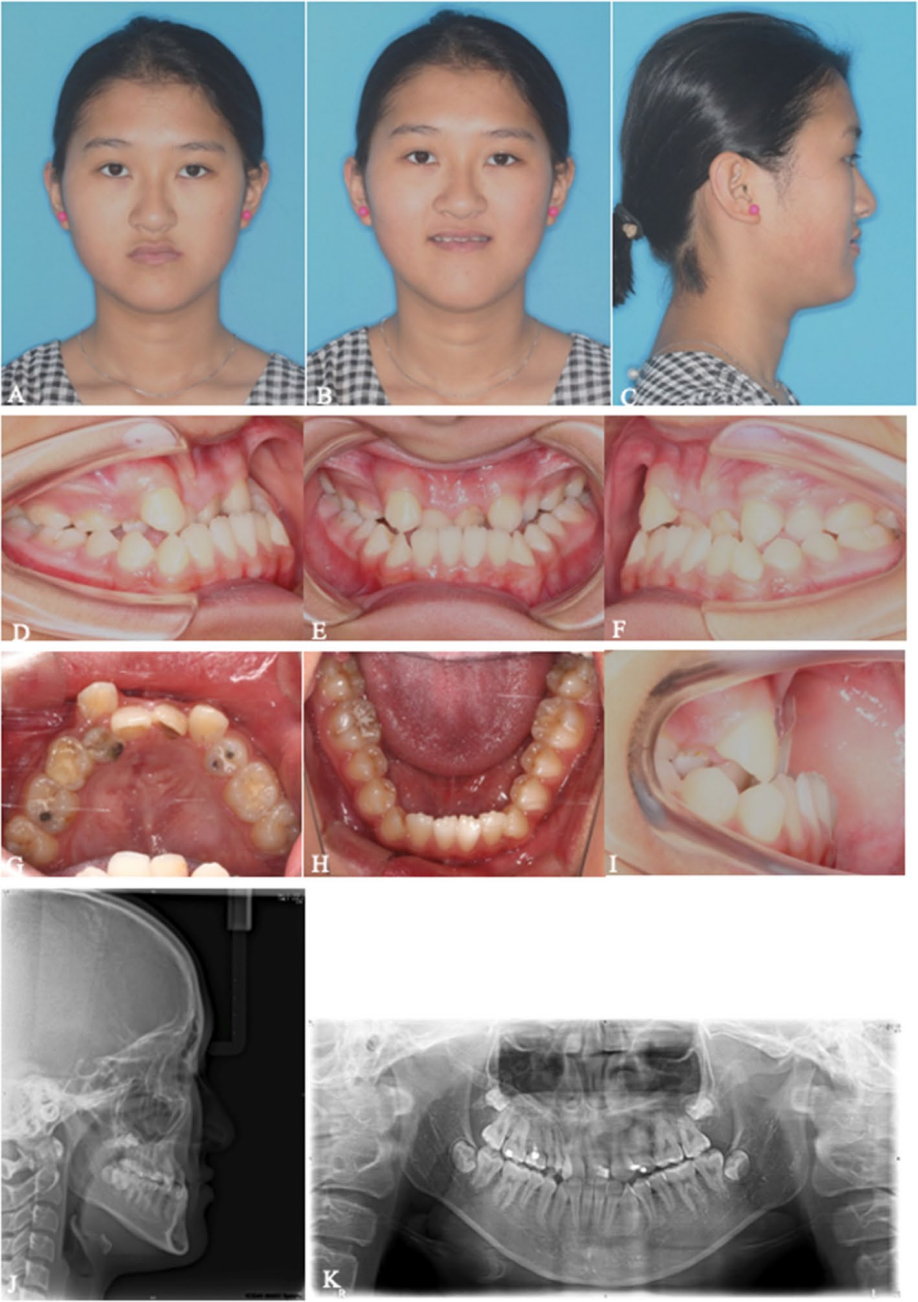
**A**-**K** Pretreatment extra- and intra-oral photographs, lateral cephalograms and panoramic of #3. A 16-year-old Chinese girl who presented with incomplete cleft palate and had been received palatoplasty in infancy, was referred to our clinic for the correction of a concave facial profile and severe dental crowding. The extraoral examination showed a midface deficiency and protruded mandible with deviation. There was an increased maxillary height on the right and an associated cant in the occlusal plane. The intraoral examination showed an anterior crossbite about 6 mm and bilateral posterior crossbite. She was missing her No.12, 15, 22, 35 and 1E was retained. No.13 was totally labial position. Severe crowding in the upper arch with no crowding in the lower arch was observed. The lower dental midline deviated 1.5 mm to the left relative to the facial midline. The cephalometric analysis showed a skeletal Class III relationship with a retrusive maxilla and a protrusive mandible. The following treatment plan was proposed: (1) No.1E extraction and palatal movement of No.13; (2) maxillary forward advancement with MASDO; (3) alignment of the malposed teeth and space were reserved for the restoration of No.15 and No.25; (4) correction of a class III malocclusion and facial asymmetry with a bimaxillary osteotomy; (5) completion to achieve tight intercuspation; (6) retention

**Fig. 9:**
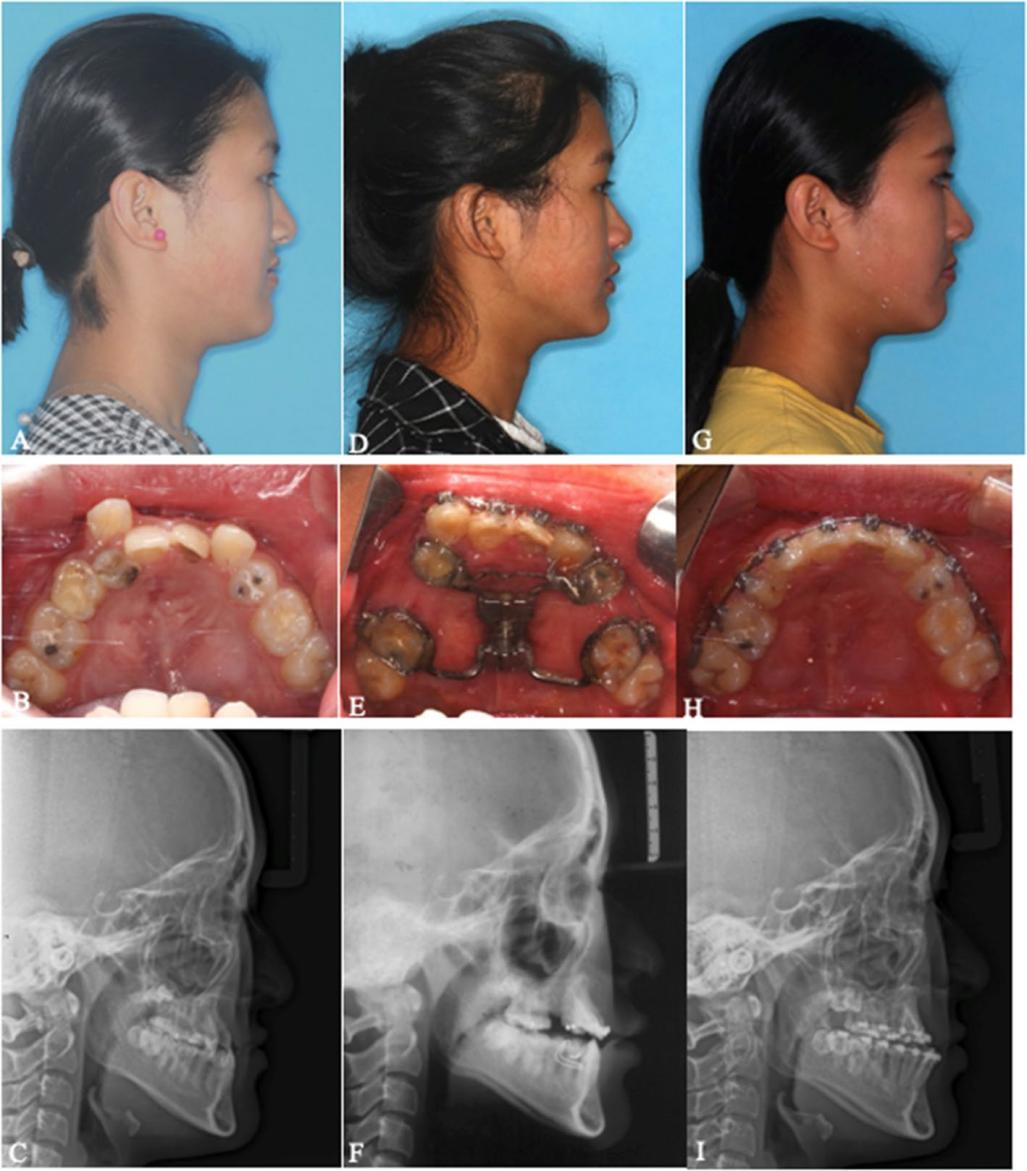
**A-C** Photographs, intraoral view and lateral cephalometric radiograph before treatment. **D-F** Photographs, intraoral view and lateral cephalometric radiograph after the consolidation period. **G-I** Photographs, intraoral view and lateral cephalometric radiograph after preoperative orthodontic treatment. MASDO was applied and the osteotomy line was drawn on the palate between the molars and premolars on both sides. One mini-screw was inserted in the planned position. Loosening of the mini-screw occurred at 12 weeks after the osteotomy. The premaxilla moved forward and the facial profile was improved after MASDO. We initially planned to alleviate dental crowding and create space for the restoration of No.15 and No.25 using the space produced by the MASDO produce. However, the patient wished to close the space without restoration, so the distracted space was subsequently closed by mesializing the molars

**Fig. 10:**
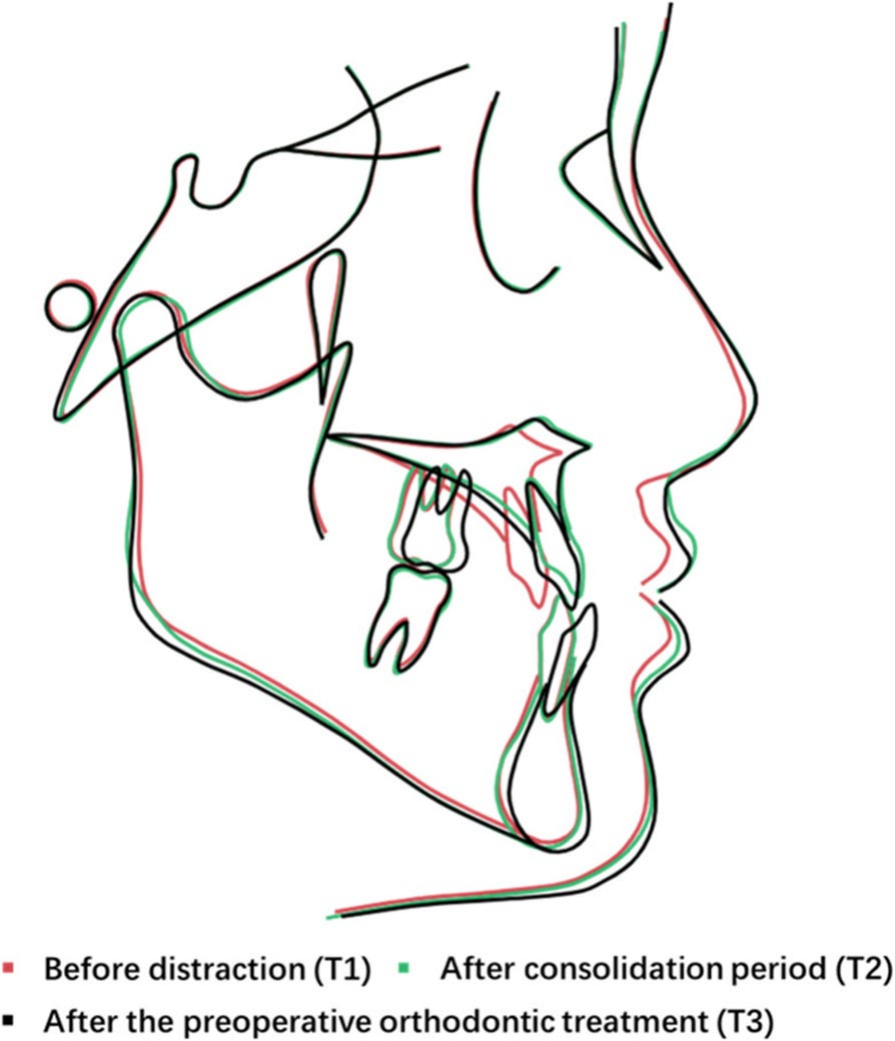
Superimposition of the profilogram on the sellanasion plane at the sella. Superimposition of cephalometric tracings of #1 made before distraction (T1), after consolidation period (T2) and after the preoperative orthodontic treatment (T3). MASDO caused significant advancement in the anterior segment of the maxilla and improved patients’ facial aesthetics with minimal relapse

**Fig. 11:**
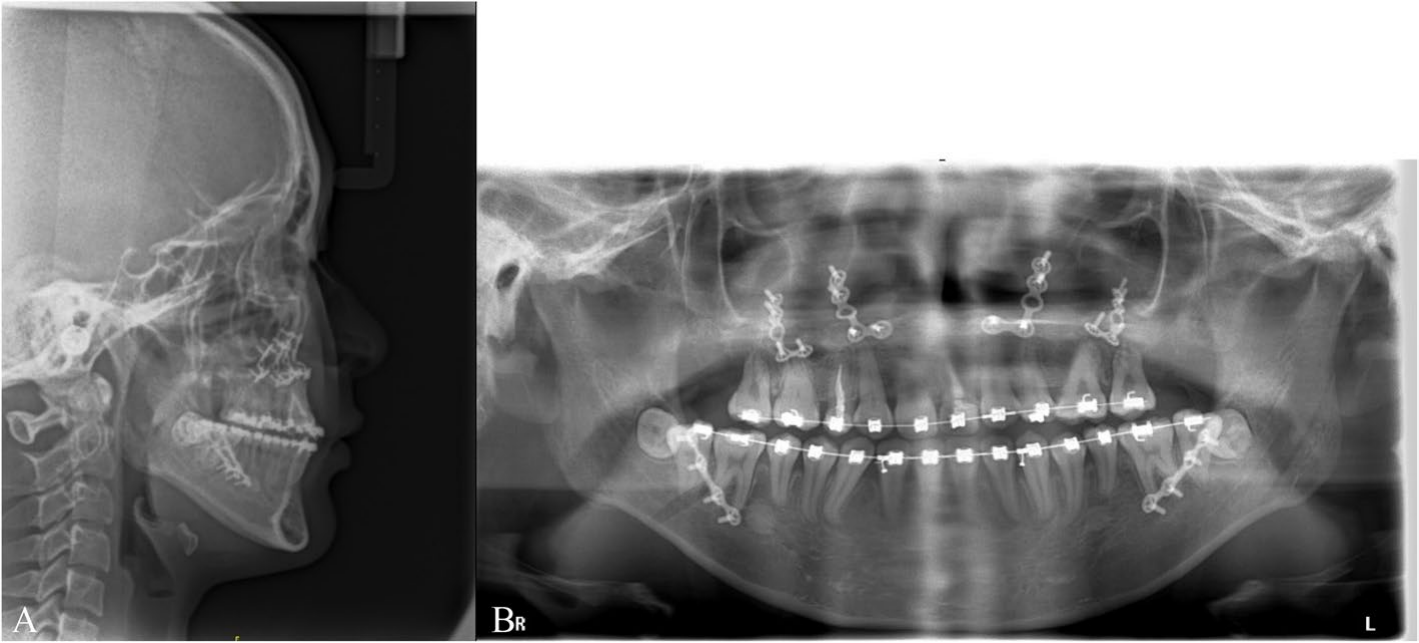
**A**, **B** Lateral cephalometric and panoramic radiographs 6 weeks after orthognathic surgery of #3. 22 months after the MASDO procedure, a bimaxillary osteotomy was performed to correct the class III malocclusion and facial asymmetry. The maxilla was advanced by 1 mm and the left side moved downward 1.5 mm to correct the canting of the occlusal plane. The amount of mandibular setback was approximately 4.0 mm on the right side and 2.5 mm on the left side

**Fig. 12:**
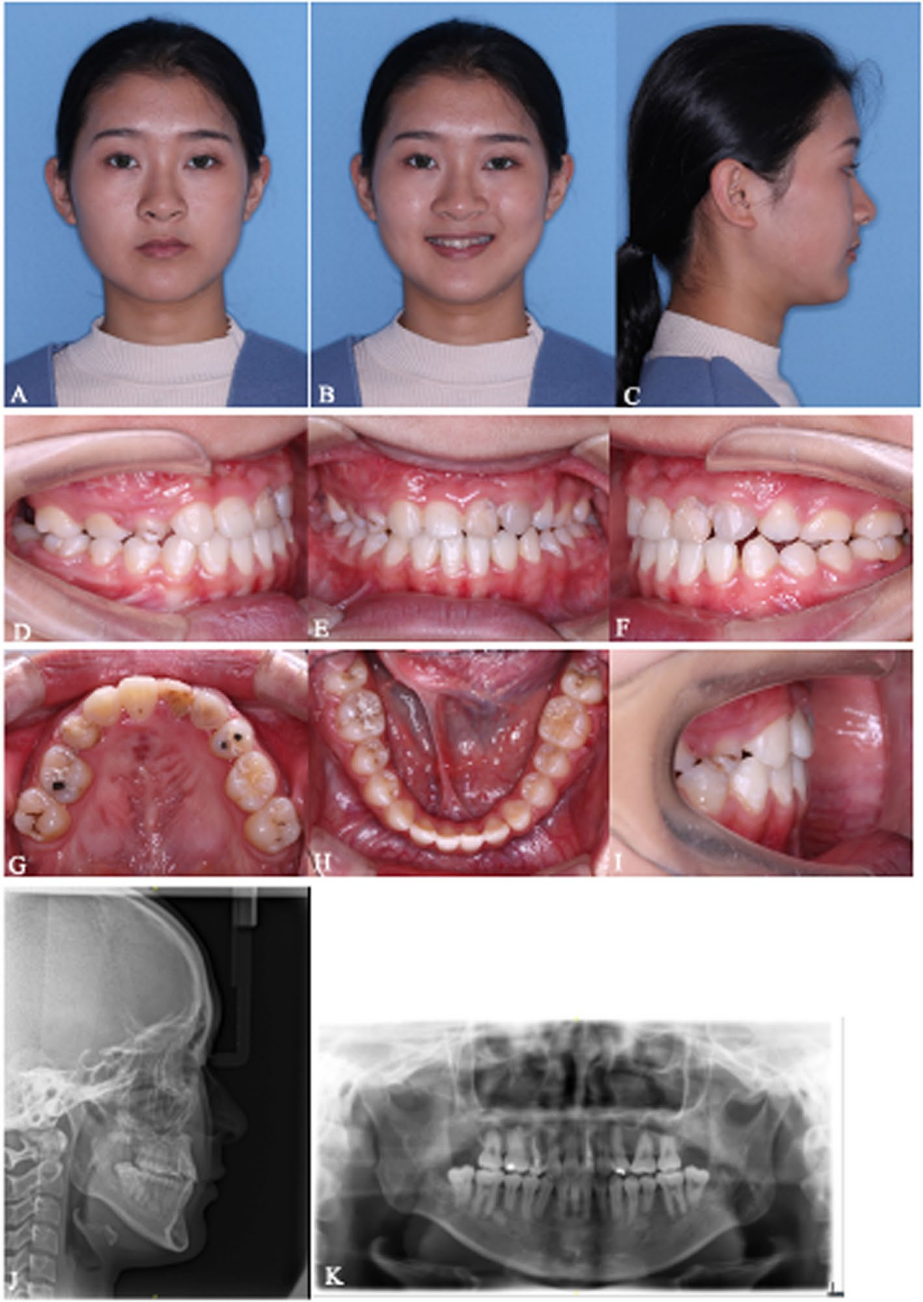
**A**-**K** After orthognathic surgery and postoperative orthodontic treatment extra- and intra-oral photographs, lateral cephalograms and panoramic of #3. The titanium plates were removed 10 months after orthognathic surgery. All appliances were removed and the final X-rays were taken 12 months after orthognathic surgery

All 6 patients successfully underwent MASDO with customized tooth-borne distractors (two patients with 2 miniscrews and four patients with 1 miniscrew) to correct the maxillary deficiency. During both the surgical and distraction periods, no serious complications, such as severe hemorrhage, infection, tooth and nerve injury or bone necrosis, were observed. No obvious pulpitis, gingival recession or abnormal dental mobility was observed among any anchorage teeth. Loosening of the mini-screw occurred in only one patient during the consolidation period. All patients received routine orthodontic treatment after MASDO and orthognathic surgery was performed in four patients. The follow-up period from T2 to T3 ranged from 22 to 26 months (mean 23.8 months) (Table 2). Two representative cases with improved facial profiles and dental alignment are shown in Figs. 3, 4, 5, 6, 7, 8, 9, 10, 11 and 12.

**Table 2:** Patient characteristics

#	Gender	Age (years)	Original Diagnosis	Miniscrew	Orthodontic Period (T3-T2) (months)	Orthognathic surgery
1	F	15	SMCP	2 (8.0 mm; Φ2.0 mm)	26	BSSRO
2	F	14	SMCP	1 (10.0 mm; Φ2.0 mm)	24	No
3	F	16	ICP	1 (8.0 mm; Φ2.0 mm)	22	LFI, BSSRO
4	F	16	ICP	1 (10.0 mm; Φ2.0 mm)	22	LFI, BSSRO
5	M	15	CSP	1 (10.0 mm; Φ2.0 mm)	24	No
6	F	15	CSP	2 (10.0 mm; Φ2.0 mm)	25	BSSRO
Avg		15.2			23.8	

*SMCP* Submucous cleft palate, *CSP* Cleft of the soft palate, *ICP* Incomplete cleft palate

### Cephalometric assessment

All the tracings and calculations were repeated by the same author six weeks after the first measurements. The intraclass correlation coefficients between the first and second measurements indicated high reliability (> 0.90). The mean values of hard tissue (skeletal and dental) and soft tissue angular and linear measurements at T1, T2, and T3 are given in Table 3. The mean changes during distraction (T1–T2), fixed orthodontic treatment (T2–T3) and the entire treatment period (T1–T3) and the significance of these mean changes are presented in Table 4.

**Table 3:** Mean values of skeletal, dental and soft tissue variables

Variables		T1		T2		T3	
		Mean	SD	Mean	SD	Mean	SD
**Skeletal**							
1	SNA (°)	75.60	2.42	79.88	2.27	79.62	2.25
2	FH⊥N–A (mm)	-6.50	3.14	-2.57	2.34	-2.73	2.36
3	SNB (°)	81.95	2.03	82.18	2.31	82.20	2.20
4	ANB (°)	-6.05	2.13	-1.95	2.38	-2.27	2.53
5	SN/ANS-PNS (°)	9.38	1.97	8.25	1.54	8.22	1.46
6	SN/Go-Gn (°)	31.95	2.17	32.65	2.21	32.38	2.334
7	CFH-ANS (mm)	43.96	2.23	43.02	2.22	42.90	2.02
8	VRL-ANS (mm)	60.28	6.06	64.26	5.76	63.92	5.62
9	CFH-A (mm)	48.85	2.28	47.82	2.16	47.82	2.19
10	VRL-A (mm)	54.58	4.83	59.24	4.89	58.75	4.88
11	VRL-B (mm)	60.52	3.81	60.96	2.94	60.80	3.34
12	VRL-Pg (mm)	61.28	3.77	62.06	2.36	61.60	3.49
**Dental**							
13	U1/ANS-PNS (°)	110.8	5.25	112.2	6.40	112.8	5.43
14	U1/SN (°)	101.7	6.66	104.3	7.35	104.9	6.21
15	CFH-U1 (mm)	67.96	3.76	67.39	3.85	68.21	4.30
16	VRL-U1 (mm)	57.44	5.26	62.89	5.32	62.51	5.46
17	CFH-U6 (mm)	63.37	1.74	62.92	1.89	63.27	2.11
18	VRL-U6 (mm)	35.88	2.23	35.66	2.22	36.32	2.22
19	Overjet (mm)	-5.47	2.97	-0.30	3.31	-1.87	2.79
20	Overbite (mm)	1.14	0.40	0.53	0.38	1.07	0.42
21	IMPA (°)	79.70	7.29	79.65	7.16	85.65	3.54
**Soft tissue**							
22	Ls-E line (mm)	-5.23	2.15	-2.02	2.93	-2.51	2.98
23	Li-E line (mm)	-0.18	2.94	-0.16	2.93	0.73	2.13
24	Nasolabial angle	75.32	8.51	88.85	4.68	88.80	5.43
25	VRL-Pn	83.57	2.88	85.31	2.94	85.66	3.05
26	VRL-Sn	69.10	1.96	73.37	2.03	73.02	2.12
27	VRL-Ss	69.04	2.58	74.32	2.91	74.05	3.44
28	VRL-Ls	73.70	3.80	77.60	3.33	77.00	3.40
29	VRL-Li	76.85	4.14	77.71	4.56	78.74	3.57
30	VRL-Si	71.84	4.62	71.66	4.82	72.32	4.84
31	VRL-Pg'	72.94	4.25	72.92	4.49	72.90	4.04

**Table 4:** Significance of mean changes of skeletal, dental, and soft tissue variables

Variables		T1-T2			T2-T3			T1-T3		
		D	SD		D	SD		D	SD	
**Skeletal**										
1	SNA (°)	4.28	0.30	< 0.001^***^	-0.27	0.10	0.083^ ns^	4.02	0.26	0.083^ ns^
2	FH⊥N–A (mm)	3.94	0.98	0.002^**^	-0.16	0.11	0.248^ ns^	3.78	0.93	0.043^*^
3	SNB (°)	0.23	0.77	> 0.999^ ns^	0.02	0.37	> 0.999^ ns^	0.25	0.92	> 0.999^ ns^
4	ANB (°)	4.10	0.75	0.002^**^	-0.32	0.34	0.248^ ns^	3.78	0.82	0.043^*^
5	SN/ANS-PNS (°)	-1.13	0.63	0.014^**^	-0.03	0.12	0.773^ ns^	-1.27	0.67	0.006^**^
6	SN/Go-Gn (°)	0.70	0.20	0.006^**^	-0.27	0.43	0.471^ ns^	0.43	0.45	0.043^*^
7	CFH-ANS (mm)	-0.93	0.40	0.009^**^	-0.13	0.36	> 0.999^ ns^	-1.06	0.49	0.009^**^
8	VRL-ANS (mm)	3.98	0.71	< 0.001^***^	-0.34	0.18	0.083^ ns^	3.64	0.81	0.083^ ns^
9	CFH-A (mm)	-1.03	0.52	0.006^**^	0.01	0.07	0.773^ ns^	-1.03	0.49	0.014^*^
10	VRL-A (mm)	4.66	0.73	< 0.001^***^	-0.49	0.17	0.083^ ns^	4.17	0.65	0.083^ ns^
11	VRL-B (mm)	0.44	1.18	0.773^ ns^	-0.16	0.74	0.773^ ns^	0.28	0.96	0.564^ ns^
12	VRL-Pg (mm)	0.78	1.61	0.564^ ns^	-0.26	1.51	0.248^ ns^	0.53	0.64	0.564^ ns^
**Dental**										
13	U1/ANS-PNS (°)	1.37	1.26	0.043^*^	0.58	2.68	0.773^ ns^	1.95	2.20	0.021^*^
14	U1/SN (°)	2.10	2.18	0.043^*^	0.60	2.65	0.773^ ns^	3.15	2.48	0.021^*^
15	CFH-U1 (mm)	-0.57	1.19	0.564^ ns^	0.82	0.95	0.248^ ns^	0.25	1.98	0.564^ ns^
16	VRL-U1 (mm)	5.45	0.66	0.009^**^	-0.37	0.90	> 0.999^ ns^	5.08	0.77	0.009^**^
17	CFH-U6 (mm)	-0.46	0.35	0.043^*^	0.35	0.24	0.149^ ns^	-0.10	0.53	0.564^ ns^
18	VRL-U6 (mm)	-0.23	0.14	0.043^*^	0.66	0.71	0.006^**^	0.44	0.83	0.471^ ns^
19	Overjet (mm)	5.17	0.79	0.021^*^	-1.56	3.93	0.773^ ns^	3.60	3.36	0.043^*^
20	Overbite (mm)	-0.61	0.46	0.009^**^	0.54	0.67	0.083^ ns^	-0.07	0.49	0.387^ ns^
21	IMPA (°)	-0.05	0.23	> 0.999^ ns^	5.00	5.35	> 0.999^ ns^	4.95	5.42	0.937^ ns^
**Soft tissue**										
22	Ls-E line (mm)	3.22	1.01	0.002^**^	-0.50	0.44	0.248^ ns^	2.72	0.97	0.043^*^
23	Li-E line (mm)	0.02	0.26	0.564^ ns^	0.90	1.49	0.248^ ns^	0.91	1.34	0.564^ ns^
24	Nasolabial angle	13.53	7.93	0.004^**^	-0.05	2.681	0.564^ ns^	13.48	7.63	0.021^*^
25	VRL-Pn	1.74	0.65	0.021^*^	0.36	0.49	0.564^ ns^	2.09	0.42	0.004^**^
26	VRL-Sn	4.27	0.86	0.002^**^	-0.36	0.25	0.248^ ns^	3.92	0.76	0.043^*^
27	VRL-Ss	5.28	1.39	0.004^**^	-0.28	0.75	0.564^ ns^	5.01	1.39	0.021^*^
28	VRL-Ls	3.90	0.91	0.002^**^	-0.61	0.47	0.248^ ns^	3.30	0.85	0.043^*^
29	VRL-Li	0.86	0.84	0.043^*^	1.03	1.06	0.248^ ns^	1.89	0.84	0.002^**^
30	VRL-Si	-0.19	0.51	0.149^ ns^	0.67	0.71	0.043^*^	0.48	0.76	0.564^ ns^
31	VRL-Pg'	-0.02	0.54	> 0.999^ ns^	-0.02	0.70	0.387^ ns^	-0.04	0.80	0.387^ ns^

*ns* non-significant

^*^minus sign values indicate a decrease

^*^p < 0.05, ^**^p < 0.01, ^***^p < *0.001*

From T1 to T2, we found that MASDO caused significant advancement in the anterior segment of the maxilla, with changes of 3.94 ± 0.94 mm (*p* < 0.05), 3.98 ± 0.71 mm (*p* < 0.05) and 4.66 ± 0.73 mm (*p* < 0.01) for FH ⊥ N–A, VRL–ANS and VRL–A, respectively. Statistical analyses also revealed increases in SNA and ANB. At the same time, significant upward movements of points ANS (CFH–ANS) and A (CFH–A) by 0.93 ± 0.40 mm (*p* < 0.05) and 1.03 ± 0.52 mm (*p* < 0.05) were observed, respectively. The maxillary plane angle (SN/ANS–PNS) decreased significantly by 1.13 ± 0.63° (*p* < 0.05).

After distraction, a significant decrease in overjet and an increase in overbite were obtained *(p* < 0.05). Anterior movements of the upper incisors (VRL–U1) were significant (*p* < 0.05). Anterior tipping of the upper incisors (U1/ANS–PNS and U1/SN) were observed (*p* < 0.05). In addition, the skeletal movement (VRL–A) was 86% (4.66 mm/5.45 mm) of the incisor movement (VRL–U1) during distraction.

Comparative evaluation between T1 and T2 showed that anterior movement of the point pro-nasale (VRL–Pn) and upper lip (Ls–E line, VRL–Sn, VRL–Ss, and VRL–Ls) was significant (*p* < 0.05). In addition, a significant increase in the nasolabial angle was measured (*p* < 0.05). However, none of the above data showed statistically significant changes between T2 and T3.

## Discussion

Among CLP patients, hypoplastic maxilla has been considered a major issue, and LFI advancement has been regarded as an ideal treatment option. However, LFI osteotomy was able to correct only the position of the maxilla but provided no space to solve the dental crowding. Furthermore, velopharyngeal function deteriorated in some patients with LFI maxillary advancement due to advancement of the posterior part of the maxilla complex [18]. Maxillary distraction following Le Fort I osteotomy has many advantages, including greater advancement, more stable long-term results and reduced negative effects on velopharyngeal competence and has therefore been used to improve maxillary hypoplasia in CLP patients [7, 8, 15, 19].

However, the hypoplastic maxilla sometimes results in arch length discrepancies and lacks space for teeth. Maxillary distraction following Le Fort I osteotomy does not change the sagittal length of the maxillary dental arch and does not resolve the dental crowding problem. Due to the interdental osteotomy at the premolar region of the upper arch, MASDO is essential to create an alveolar bone space for tooth alignment and simultaneously correct maxillary hypoplasia [9, 20].

In the present study, all six patients had an anterior crossbite and severe dental crowding due to skeletal sagittal deficiency and space shortages in the dental arch. At least two upper premolars usually need to be extracted to create space to alleviate dental crowding, which results in unmatched numbers of teeth in the upper and lower arches. Here, we utilized MASDO using a miniscrew assisted tooth-borne distractor to advance the anterior maxillary segment. The results show that the premaxilla moved forward, the length of the palatal plane and upper arch increased, and sufficient space was gained to align the crowding teeth in all six patients. In addition to bone advancement, the surrounding soft tissue was simultaneously regenerated and moved forward, thus improving the patients’ facial aesthetics. Our measurements of facial convexity and the nasolabial angle revealed that the protrusion of facial convexity was significantly ameliorated and that the nasolabial angle also increased following advancement of the nasal base and uplifting of the pronasale.

MASDO with different types of extraoral and intraoral distractors has been performed. Extraoral distractors have the volume for multidirectional maxillary advancement, and the vectors can be changed during the distraction period. However, many patients have difficulties with extraoral devices, primarily due to unfavorable aesthetics [15]. Intraoral appliances can be maintained during the consolidation period to prevent relapse because patients tolerate them with fewer psychosocial problems than with extraoral appliances. Intraoral appliances are classified into bone-borne and tooth-borne distractors [14, 17, 21, 22]. Bone-borne intraoral appliances were introduced to provide effective distraction without dental effects, but because the appliances contained four mini-screws, it was more difficult to apply, and the risk of infection was relatively high. Tooth-borne intraoral appliances can be applied to distract alveolar bone by 8 to 10 mm. However, such appliances are expected to increase the load onto the anterior teeth and may exert a significant dental effect [4, 18].

In the present study, a new tooth-borne device was used for distraction. This new device used a miniscrew (skeletal anchorage) to advance the premaxilla and bands (tooth anchorage) to support the posterior segment of the maxilla. The skeletal movement (VRL–A) was 86% (4.66 mm/5.45 mm) of the incisor movement (VRL–U1) during distraction. This ratio was equal to that in a study performed by Block et al. with a bone-borne device [5]. Ho et al. and Cakmak et al. reported skeletal movement ratios of 70 and 62% with a tooth-borne device [17, 23]. This new tooth-borne distractor achieved the same effectiveness of distraction as bone-borne distractors while decreasing the load onto the anterior teeth. Furthermore, the distractor used in the present study had the advantages of less trauma, easy fabrication, minimal costs and good patient tolerance than a bone-borne distractor.

With regard to skeletal changes after MASDO, our results from cephalometric analysis revealed that the segments advanced forward and slightly upward. Significant upward movements of points ANS (CFH–ANS) and A (CFH–A) by 0.93 ± 0.40 mm and 1.03 ± 0.52 mm were observed, respectively. The maxillary plane angle (SN/ANS–PNS) decreased significantly by 1.13 ± 0.63° (*p* < 0.05). These data implied counterclockwise rotation of the maxillary anterior segment after MASDO. The distraction appliance was a tooth-borne device, and the line of action of force was below the center of resistance of the premaxilla. Thus, anterior rotation of the premaxilla was inevitable [17]. This phenomenon has been found in several clinical studies applying a tooth-borne distraction device [1, 14, 23]. To achieve sophisticated three-dimensional positioning of the anterior segment using tooth-borne distractors, face mask-type protraction headgear may be used to assist in controlling the traction direction of the premaxilla in future cases, and careful observation and continuous anterior traction with elastics during both the distraction and consolidation periods may help achieve precise positioning of the maxilla [9, 24].

Relapses of maxillary distraction following Le Fort I osteotomy in the long term were reported to be minimal due to gradual advancement of the maxilla [25]. Although some published studies have described the efficacy of MASDO in managing cleft maxillary hypoplasia, the long-term stability of MASDO has rarely been reported. Richardson et al. found a relapse rate of 4.76% in SNA 1 to 4 years after MASDO with tooth-borne distractors [24]. Kanzaki et al. reported that the relapse rates of the maxilla 1 year after distraction were 21.2% in the MASDO group and 13.4% in the rigid external distraction (RED) group, with no significant difference between the MASDO and RED groups [9]. Tanikawa et al. reported that the median percentage of relapse at 1 year after MASDO was 10% for the A-McNamara value. They found that patients with increased relapse showed significant intraoperative counterclockwise rotation of the maxilla compared with patients with smaller relapse [22].

In the present study, no obvious relapse was observed after at least 22 months of follow-up. Both favorable skeletal and soft tissue changes in the midface after MASDO were stable. We presumed that MASDO extended not only to skeletal tissues but also to soft tissues in a direction opposite to that of soft tissue tension during the distraction period. This soft tissue expansion, which took approximately 2 weeks, would release soft tissue tension and increase postoperative stability. Cheung et al. reported that the magnitude of relapse tended to be correlated with the magnitude of maxillary advancement [26]. In our study, the distraction distance was designed to be 7 mm, which was not a large amount of advancement and reduced the recurrence rate to some extent, thus increasing postoperative stability.

It's worth noting that many measurements of sagittal correction during T1-T2 showed posterior movement during T2-T3. These included SNA, ANB, Overjet, Ls-E line, and VRL to ANS, A, U1, Sn, Ss, and Ls. Although the T2-T3 changes were not significant, they may suggest a trend toward slight relapse. It was noted that 4 patients (2 clefts of the soft palate and 2 incomplete cleft palates) in our study had palatal scar from a previous palatal cleft repair. This seems to be the main factor that contributed to relapse [3]. We could observe a small increase in SNB, so the continuous growth of the mandible during the postoperative period might also result in the reduction of ANB [24]. Overjet reduction occurred mainly in patients who underwent orthognathic surgery because of lower incisors decompensation during postoperative orthodontic treatment. The lower incisors were proclinated during decompensation, thus increasing overjet. Due to the relapse problems encountered, Cheung advocated overcorrection of the maxilla to overcome the skeletal relapse. However, considering that increasing maxillary advancement may also increase the degree of relapse and increase the possibility of counterclockwise rotation of the maxillary anterior segment, a larger sample size would be needed to confirm the true extent of the amount of overcorrection [22].

When a palatal device is used as a miniscrew assisted tooth-borne distractor, the most difficult challenge is determining the location of miniscrew, because there are anatomical disadvantages in palatal form for CLP patients, including short palatal dimension and underdeveloped small, thin palatal bones [12]. The position chosen to secure the miniscrew should be determined by means of three-dimensional computed tomography so that we could confirm the palatal bone shape and thickness, the location of the palatal foramen and the position of incisor’s root. On the basis of the assessment, if there is no enough space or thick bone to support the miniscrew, a tooth-borne distractor without miniscrew may be more suitable. In this study, the loosening rate of miniscrew was relatively low, only patient #3 experienced miniscrew loosening during the consolidation period (Fig. 9). In this patient, the length and width of the maxillary anterior segmental were small due to the absence of lateral incisors. The implant site of the miniscrew was relatively close to the anterior teeth, so we chose a shorter miniscrew (8.0 mm; diameter:2.0 mm) to avoid being too close to the roots of upper incisors. Shorter miniscrew and thinner palatal bone due to cleft palate may be responsible for the loosening of miniscrew.

CLP patients typically present with symptoms of maxillary hypoplasia at an early age, which can seriously affect their facial aesthetics, oral function and psychological health, and may require early surgical intervention. However, little is known about the effects of orthognathic surgical procedures on subsequent facial growth when they are performed in growing pediatric and adolescent patients. Maxillary growth spurts have been reported for girls between 10 and 12 years of age. Boys generally experience their adolescent spurt 1 to 3 years later than girls [27]. In the present study, the subjects were 14–16 years old and CVMS IV-V; thus, we think that the subjects have passed the growth spurt and MASDO has very little influence on the development of the maxilla. We believe that these teenagers are suitable for MASDO because they can experience prominent benefits, including improvements in maxillary hypoplasia, facial profiles and dental occlusion, at relatively young ages. However, more studies are warranted to explore whether MASDO affects later craniomaxillofacial development in young people.

The main limitation of this study was that the sample size used was quite small, and this could be attributed to the low prevalence of cleft deformities. CLP patients without an alveolar cleft are not common in Shanghai and this restricted the sample size considerably. Future studies incorporating larger sample sizes are necessary. Ideally, an RCT should be designed to confirm the optimal treatment procedure if ethically permissible. Furthermore, more studies are needed to determine the effectiveness and stability of the procedure for cleft maxillary hypoplasia with alveolar cleft. CBCT can be used in future studies to increase the accuracy of measurements. Symmetry analysis of MASDO and the measurements of pharyngeal volume can be performed using CBCT. However, we believe that the present study should be considered as a preliminary study examining the feasibility of using the miniscrew assisted tooth-borne distractor for treating patient with cleft-related maxillary hypoplasia.

## Conclusions

MASDO using a miniscrew assisted tooth-borne distractor presented significant maxillary advancement, less dental effect and minimal relapse, which can be used for cleft maxillary hypoplasia without an alveolar cleft. Additionally, MASDO allows us to address the problem of cleft maxillary hypoplasia at a younger age. Furthermore, due to the limited amount of material to be implanted, this distractor has affordability, a simple design, and several advantages over the current armamentarium of bone-borne devices and tooth-borne designs.

## Data Availability

The datasets used and/or analysed during the current study available from the corresponding author on reasonable request.

## Abbreviations

CLP: Cleft lip and palate
VPI: Velopharyngeal insufficiency
LFI: LeFort I osteotomy
DO: Distraction osteogenesis
MASDO: Maxillary anterior segmental distraction osteogenesis
CVMS: Cervical vertebral maturation Stage
CFH: Constructed Frankfurt Horizontal
VRL: Vertical Reference Line
BSSRO: Bilateral sagittal split ramus osteotomy
RCT: Randomized controlled trial
CBCT: Cone beam Computer Tomography
